# Active modulation of the calcifying fluid carbonate chemistry (δ^11^B, B/Ca) and seasonally invariant coral calcification at sub-tropical limits

**DOI:** 10.1038/s41598-017-14066-9

**Published:** 2017-10-23

**Authors:** Claire L. Ross, James L. Falter, Malcolm T. McCulloch

**Affiliations:** 1 Oceans Institute and School of Earth Sciences, The University of Western Australia, Perth, Australia; 2 Australian Research Council Centre of Excellence for Coral Reef Studies, The University of Western Australia, Perth, Australia

## Abstract

Coral calcification is dependent on both the supply of dissolved inorganic carbon (DIC) and the up-regulation of pH in the calcifying fluid (cf). Using geochemical proxies (δ^11^B, B/Ca, Sr/Ca, Li/Mg), we show seasonal changes in the pH_cf_ and DIC_cf_ for *Acropora yongei* and *Pocillopora damicornis* growing *in-situ* at Rottnest Island (32°S) in Western Australia. Changes in pH_cf_ range from 8.38 in summer to 8.60 in winter, while DIC_cf_ is 25 to 30% higher during summer compared to winter (×1.5 to ×2 seawater). Thus, both variables are up-regulated well above seawater values and are seasonally out of phase with one another. The net effect of this counter-cyclical behaviour between DIC_cf_ and pH_cf_ is that the aragonite saturation state of the calcifying fluid (Ω_cf_) is elevated ~4 times above seawater values and is ~25 to 40% higher during winter compared to summer. Thus, these corals control the chemical composition of the calcifying fluid to help sustain near-constant year-round calcification rates, despite a seasonal seawater temperature range from just ~19° to 24 °C. The ability of corals to up-regulate Ω_cf_ is a key mechanism to optimise biomineralization, and is thus critical for the future of coral calcification under high CO_2_ conditions.

## Introduction

Coral reefs face an uncertain future due to increasing seawater temperatures and ocean acidification resulting from CO_2_-driven climate change^1,2^. Rising ocean temperatures are expected to lead to more frequent mass coral bleaching events, defined as a loss of the endosymbiotic dinoflagellates (zooxanthellae) from the coral host^3–5^. While corals have demonstrated the capacity to adapt to long-term changes in climate, they are still extremely vulnerable to abrupt warming events (i.e., weeks to months) as evident, for example, by the mass bleaching events that often follow El Niño-Southern Oscillation (ENSO) driven warming events^6,7^. Additionally, evidence suggests^8–10^ that declining seawater pH will cause the growth rates of important marine calcifiers, such as hermatypic corals, to slow down. The effect of declining pH on the calcification process is however still in question, as corals possess the physiological mechanisms to partially resist or limit the effects of ocean acidification on the bio-calcification process^11–16^. They accomplish this through the up-regulation of the pH of the calcifying fluid (pH_cf_); a process that likely occurs via active ionic exchange of Ca^2+^ with H^+^ via Ca-ATPase^17,18^ ‘pumps’ at the site of calcification^11–16^. This in turn helps to elevate the aragonite saturation state in the calcifying fluid (Ω_cf_), a key requirement for the formation of their calcium carbonate (CaCO_3_) skeletons^11–16,19,20^. In addition to up-regulating pH_cf_, corals supply the calcifying fluid with metabolically generated dissolved inorganic carbon (DIC)^18^. This raises the activity of carbonate ions within the calcifying fluid ([CO_3_^2−^]_cf_) and, therefore, increases Ω_cf_^21^. Thus, corals manipulate rates of aragonite precipitation by actively elevating both pH_cf_ and DIC_cf_^21^.

Despite the importance of internal carbonate chemistry in determining rates of mineral precipitation, temperature is still the main factor influencing the rates of coral growth^22–24^. This is due to both the strong temperature-dependent rate kinetics of aragonite precipitation^25^ as well as the sensitivity of coral physiology to extremes in temperature^6,26^. For example, calcification rates in tropical corals are generally thought to follow a Gaussian–shaped curve whereby calcification increases as temperature increases until an optimum is reached; after this maximum, rates decline with increasing temperature^7,24,27^. Light is also an important driver of coral calcification on both diurnal and seasonal timescales, with rates of carbon fixation by the zooxanthellae symbiont being light dependent^18,28,29^. Therefore, it is not surprising that higher rates of coral calcification are generally found in summer compared to winter^24,30–35^. These findings^24,30–35^ are consistent with the assumption that during the summer there are higher rates of metabolically-derived carbon supply to the coral^18^ and enhanced temperature-driven precipitation kinetics^25^.

A recent study by Ross *et al*.^36^, however, found that calcification rates for the reef-building species *Acropora yongei* and *Pocillopora damicornis* growing offshore Western Australia (Rottnest Island, 32°S) exhibited minimal seasonality and, in fact, exhibited similar or even higher rates in winter. The authors suggested that this uncharacteristic seasonal pattern in calcification rates was due to increased nutrient uptake in winter^36–38^ and/or a possible sub-lethal stress response in summer^36,39^. Another possibility, which is explored herein, is that corals physiologically manipulate the chemistry of their calcifying fluid to enhance rates of calcification. Earlier studies have demonstrated that the biological mediation of pH up-regulation can modulate rates of calcification^11,13,14,16^. However, our ability to infer both aspects of the carbonate chemistry (i.e., pH and DIC) under *in-situ* conditions has only recently become possible via measuring the boron isotopic composition (δ^11^B)^40–42^ and elemental abundance of boron (B/Ca)^43^ in coral skeletons. Furthermore, while other methods of interrogating the carbonate chemistry of the calcifying fluid have been developed and provide some informative results^14,15,19,28,44^, they generally must be conducted under tightly constrained laboratory conditions that differ greatly from the *in-situ* ‘natural’ reef habitats in which corals grow. Nonetheless, previous studies have shown that estimates of internal coral pH_cf_ derived from geochemical tracers^12,13,40,45^ are consistent with more direct measurements^14,19,44^, affirming that boron isotopes are providing unbiased measurements of pH at the site of calcification. With these new developments^12,21,40,45^, we can now determine how the carbonate chemistry of the calcifying fluid (pH_cf_, DIC_cf_, Ω_cf_) responds to natural and seasonally varying changes in light, temperature and seawater pH. We show that quantifying these relationships is critical to understanding and predicting how coral growth will respond to man-made climate change under real-world conditions.

Here we examine seasonal changes in the carbonate chemistry of the calcifying fluid (pH_cf_, DIC_cf_, and Ω_cf_) for branching corals, *Acropora yongei* and *Pocillopora damicornis*, sampled every 1 to 2 months for three summers and two winters in Salmon Bay, Rottnest Island (32°S), Western Australia (WA). Boron isotopic compositions (δ^11^B) are used as a proxy for pH_cf_ and are combined with skeletal B/Ca ratios to determine [CO_3_^2−^]_cf_, and hence DIC_cf_. We show that corals in this sub-tropical environment seasonally elevate Ω_cf_ to levels that are ~4 times higher than ambient seawater and ~25% higher in winter compared to summer. We also find enhanced rates of skeletal precipitation relative to that expected from inorganic rate kinetics^25^; thus emphasizing the ability of corals to manipulate their internal carbonate chemistry to promote biomineralization.

## Results

### Coral habitat

Monthly average water temperatures at Salmon Bay ranged from 19.3° to 23.7 °C during this study period, giving a seasonal range of 4.4 °C (Supplementary Fig. S1). Monthly mean light levels increased from a minimum of just 15 mol m^−2^ d^−1^ in winter to a maximum of 48 mol m^−2^ d^−1^ in summer (Supplementary Table S1). Weekly average measured seawater pH_T_ at Rottnest Island showed minimal variability (<0.05 pH units) between summer (January 2014) and winter (July 2014; see Supplementary Fig. S2). The definitions for all physical and biogeochemical variables are provided in Table 1.

**Table 1:** Nomenclature. Definition of variables used in this paper.

Variable	Units	Description
δ^11^B	‰	Boron isotope
pH_cf_	—	pH of the calcifying fluid
pH_T_	—	pH on the total hydrogen ion scale
pH_sw_	—	pH of the seawater
DIC_cf_	µmol kg^−1^	Dissolved inorganic carbon in the calcifying fluid
DIC_sw_	µmol kg^−1^	Dissolved inorganic carbon in the seawater
Ω_cf_	—	Aragonite saturation state of the calcifying fluid
Ω_sw_	—	Aragonite saturation state of the seawater
T	°C	Temperature
B/Ca	mmol mol^−1^	Boron to calcium ratio
Li/Mg	mmol mol^−1^	Lithium to magnesium ratio
Sr/Ca	mmol mol^−1^	Strontium to calcium ratio

### Internal skeletal carbonate chemistry

**Figure 1:**
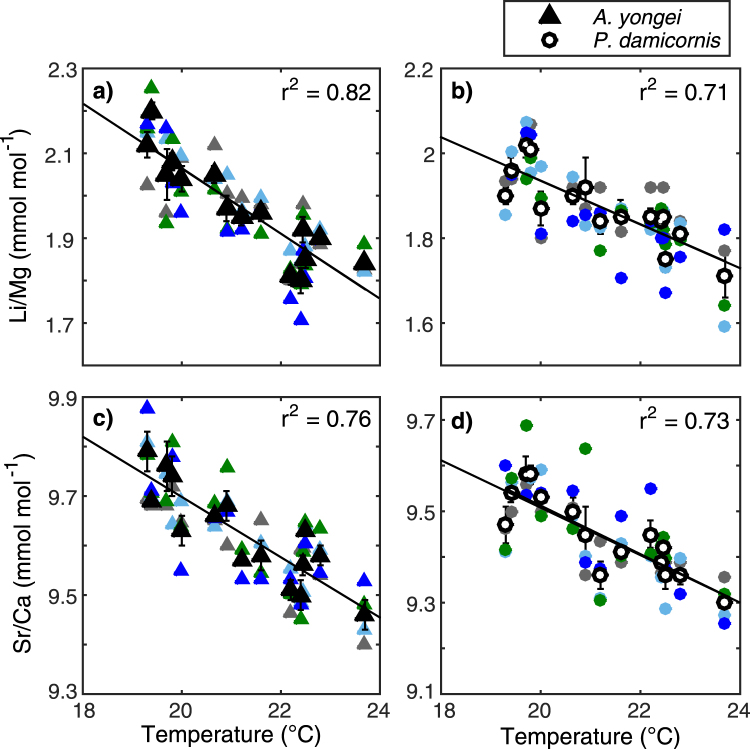
Measured Li/Mg and Sr/Ca in corals at Rottnest Island plotted against seawater temperature. (**a,b**) Li/Mg plotted against temperature with regression equation Li/Mg = −0.08T_sw_ + 3.63 for *Acropora yongei* and Li/Mg = −0.05T_sw_ + 2.99 for *Pocillopora damicornis* (**c,d**) Sr/Ca plotted against temperature with regression equation Sr/Ca = −0.061T_sw_ + 10.92 for *Acropora yongei*, and Sr/Ca = −0.053T_sw_ + 10.57 for *Pocillopora damicornis*. Coloured symbols represent each colony while the black symbols with lines denote the mean (±1 SE; *n* = 4) for each time point.

Coral skeletal Li/Mg and Sr/Ca ratios show significant inverse relationships with seasonal increases in temperature for both *A. yongei* (*r* ^2^ = 0.82, *r* ^2^ = 0.76, respectively, *p* < 0.001 for both; Fig. 1) and for *P. damicornis* (*r* ^2^ = 0.71, *r* ^2^ = 0.73, respectively, *p* < 0.001 for both; Fig. 1; Supplementary Table S2). Thus, the geochemical composition of the apical section of the coral skeleton analysed confirms that coral skeletal growth was the same time as when ambient seawater chemistry was measured. This excellent agreement is further supported by earlier measurements^36^ showing that these coral colonies grew ~4 mm month^−1^.

**Figure 2:**
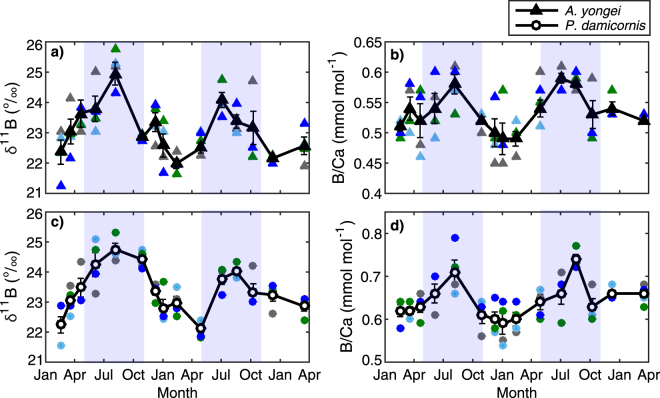
Seasonal changes in the boron isotopic signature and boron to calcium ratio (B/Ca) of corals at Rottnest Island. (**a,b**) Seasonal time-series of δ^11^B (‰) and (**c,d**) boron to calcium ratios (B/Ca) for all four colonies sampled of *Acropora yongei* and *Pocillopora damicornis*. Coloured symbols represent each colony while the black symbols with lines denote the mean ± 1 SE (*n* = 4) for each time point. Light blue shading denotes winter and unshaded areas denote summer, defined based on seasonal changes in temperature and light.

**Figure 3:**
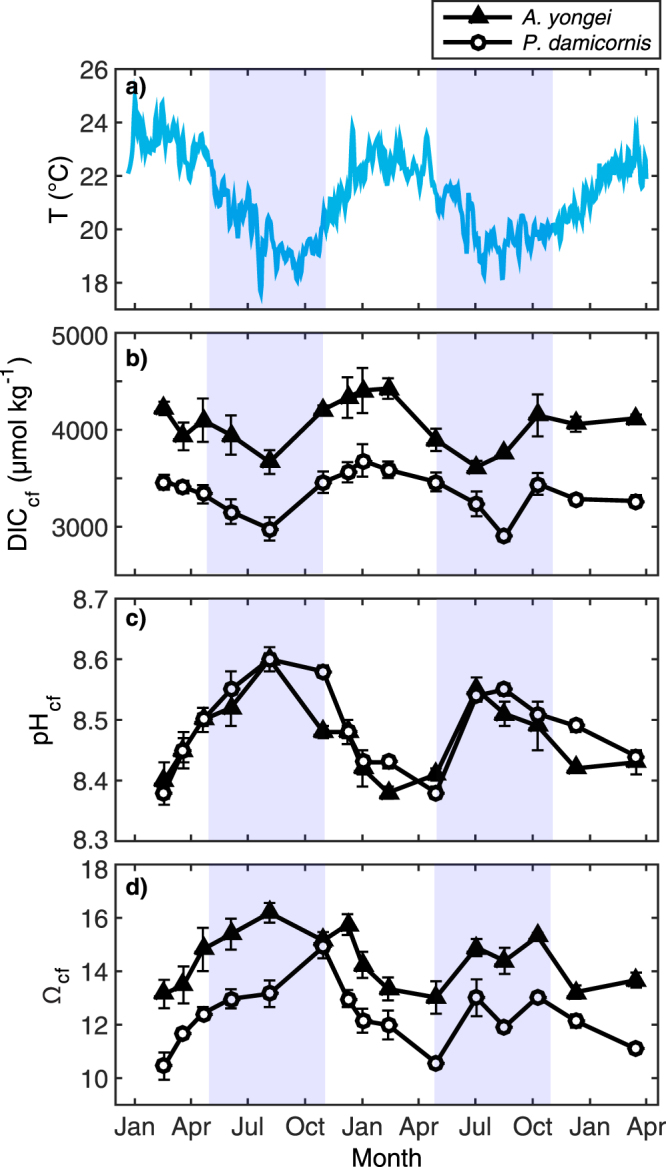
Time-series of seasonal changes in seawater temperature and calcifying fluid parameters (DIC_cf_, pH_cf_, Ω_cf_). (**a**) Seawater temperature (**b**) pH_cf_, (**c**) predicted dissolved inorganic carbon (DIC_cf_), and (**d**) Ω_cf_ for coral the species *Acropora yongei* and *Pocillopora damicornis* averaged (±1 SE) over each growth period. Light blue shading denotes winter and unshaded areas denote summer, defined based on seasonal changes in temperature and light.

Seasonal δ^11^B varies by up to 2.7‰ between the warmest month (23.7 °C) and the coolest month (19.3 °C), ranging from a minimum of 22.0‰ in February for both species to a maximum of 24.9‰ in August for *A. yongei* and 24.8‰ for *P. damicornis* (Fig. 2a,b; Supplementary Table S2). These ranges in δ^11^B correspond to seasonal variations in derived pH_cf_ of 8.38 (summer) to 8.60 (winter) for both *A. yongei* and *P. damicornis* (Fig. 3) with a lower ΔpH_cf_ of 0.28 (ΔpH_cf_ = pH_cf_ − pH_sw_) corresponding to warmer seawater temperatures and a higher ΔpH_cf_ of 0.50 corresponding to cooler seawater temperatures (Fig. 3). Skeletal ratios of boron to calcium (B/Ca) range from 0.49 to 0.59 mmol mol^−1^ for *A. yongei* and 0.59 to 0.74 mmol mol^−1^ for *P. damicornis* (Fig. 2c,d; Supplementary Table S2). These B/Ca ratios correspond to carbonate ion concentrations within the calcifying fluid [CO_3_^2−^]_cf_ of from 806 to 973 µmol kg^−1^ for *A. yongei*, and 645 to 886 µmol kg^−1^ for *P. damicornis* (Supplementary Table S3) and are ~20 to 40% higher at their peak in winter compared to their low in summer. These values fall within the range of other tropical corals as reported using carbonate-sensitive microelectrodes under laboratory conditions (600 to 1550 µmol kg^−1^)^44^.

**Figure 4:**
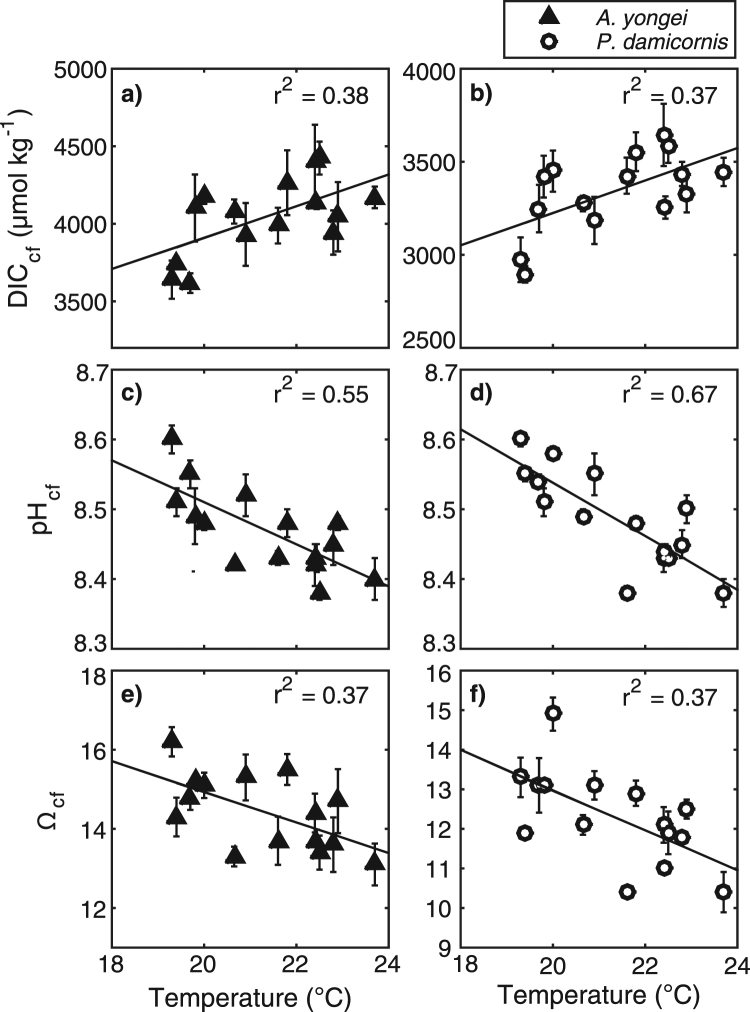
Relationships between calcifying fluid parameters versus seawater temperature. (**a,b**) Seasonal changes in DIC_cf_, (**c,d**) pH_cf_, and (**e,f**) Ω_cf_ with seawater temperatures for *Acropora yongei* and *Pocillopora damicornis* averaged (±1 SE) over each growth period.

The DIC_cf_ derived from the B/Ca ratio proxy is 1.5 to 2 times higher than ambient seawater and is positively correlated with seasonal changes in seawater temperature (*r* ^2^ = 0.38, *p* = 0.015 for *A. yongei* and *r* ^2^ = 0.37, *p* = 0.017 for *P. damicornis*; Fig. 4). The DIC_cf_ in both coral species is also 25 to 30% higher in summer compared to winter (4520 µmol kg^−1^ in summer vs. 3620 µmol kg^−1^ in winter for *A. yongei* and 3700 µmol kg^−1^ in summer vs. ~2900 µmol kg^−1^ in winter for *P. damicornis*; Fig. 3b). There is a counter-cyclical relationship between DIC_cf_ and pH_cf_ such that seasonal changes in DIC_cf_ are negatively correlated with seasonal changes in pH_cf_ (*r* ^2^ = 0.64, *p* = 0.002 for *A. yongei* and *r* ^2^ = 0.39, *p* = 0.01 for *P. damicornis*; Supplementary Fig. S3). As a result of this inverse relationship between DIC_cf_ and pH_cf_, the highest Ω_cf_ occurs in winter (16.2 for *A. yongei* and 14.9 for *P. damicornis*; Fig. 3d) and lowest in summer (13.0 for *A. yongei* and 10.4 for *P. damicornis*; Fig. 3d). Thus, Ω_cf_ is negatively correlated with seasonal changes in temperature (*r* ^2^ = 0.37, *p* = 0.015 and *r* ^2^ = 0.37, *p* = 0.02 for *A. yongei* and *P. damicornis*, respectively; Fig. 4). Due to the up-regulation of both DIC_cf_ and pH_cf_, Ω_cf_ is roughly 3.5 to 5 times higher than the mean annual seawater aragonite saturation state (Ω_sw_ ≈ 3.2) depending on taxa and season.

### Coral growth rates

Results from Ross *et al*.^36^ demonstrated that calcification rates generally deviated from their long-term (>1 year) average growth rates of 1.6 mg cm^−2^ d^−1^ for *A. yongei* and 0.67 mg cm^−2^ d^−1^ for *P. damicornis* by just ± 20% to ± 30% over the 18-month period, respectively (Table 2)^36^. These calcification rates were either negatively correlated with temperature for *P. damicornis* (*r* ^2^ = 0.45) or showed little or no seasonal coherency for *A. yongei* (i.e., no correlation with temperature *r* ^2^ = 0.015)^36^. Thus, calcification rates exhibited unexpected seasonal patterns whereby they were, on average, similar across seasons for *A. yongei*, and slighly higher in winter compared to summer for *P. damicornis*.

**Table 2:** Coral calcification rates at Rottnest Island. Seasonal changes in rates of calcification (mg cm^−2^ d^−1^; mean ± SE) for coral species (**a**) *Acropora yongei*, and (**b**) *Pocillopora damicornis* ^36^. Italic denotes winter and roman areas denote summer.

Species	Summer 2013		*Winter 2013*				Summer 2014			*Winter 2014*	Ref
	Feb	Mar	*Apr*	*Jun*	*Aug*	*Oct*	Dec	Jan	Feb	*Apr*	
*Acropora yongei*	1.54 ± 0.28	1.46 ± 0.15	2.02 ± 0.14	1.76 ± 0.12	1.61 ± 0.05	1.66 ± 0.07	1.79 ± 0.07	1.67 ± 0.06	1.33 ± 0.09	1.25 ± 0.03	36
*Pocillopora damicornis*	0.60 ± 0.12	0.47 ± 0.10	0.84 ± 0.10	0.77 ± 0.07	0.79 ± 0.11	0.90 ± 0.06	0.81 ± 0.12	0.54 ± 0.11	0.30 ± 0.07	0.55 ± 0.03	36

## Discussion

Here we show that corals living in a sub-tropical environment have the ability to modulate rates of calcification by seasonally counter-regulating pH_cf_ and DIC_cf_ to elevate Ω_cf_. The ability to infer coral pH_cf_ from δ^11^B isotopic measurements is supported by measurements from microelectrodes^19,28^ and pH-sensitive dyes^13,14,46^. For example, previous studies^13,14^ have shown that pH_cf_ up-regulation is ~0.6 to 2 units above seawater values in the light. Results from δ^11^B-derived pH_cf_ generally fall within this range and are broadly consistent^46^ with these measurements (~0.3 to 0.6 pH units above seawater)^11–13,16,20^, given that they are integrated over multiple weeks of biomineralization. Additional variability between methods (i.e., geochemical proxies and direct measurements) may also result from the different species used and the conditions under which the measurements are conducted^47^. More recent, albeit limited, measurements of the carbonate ion concentration using microelectrodes report values of 600 to 1550 µmol kg^−1^ in the calcifying fluid^44^. These instantaneous measurements performed under laboratory conditions, while providing some informative results, have significant limitations. For instance, microelectrode measurements are currently unable to determine the dynamic seasonal interactions between components of the coral calcifying fluid carbonate chemistry (pH_cf_, DIC_cf_, Ω_cf_) documented here under naturally fluctuating conditions. This is due to the limitations of existing technology, as measurements must be conducted using separate probes for pH_cf_ and [CO_3_^2−^]_cf_ and under highly controlled laboratory conditions. Thus, our findings highlight the importance of δ^11^B and B/Ca proxies as a method for inferring the internal carbonate chemistry in corals under real-world conditions and over longer (i.e., seasonal) time scales.

Laboratory experiments have nevertheless demonstrated that coral pH_cf_ typically shows a relatively consistent linear and muted response to changes in pH_sw_, such that changes in coral pH_cf_ are usually equal to approximately one-third to one-half of those in pH_sw_^12,14,47–51^. Thus, according to the results of those experiments, seasonal changes in coral pH_cf_ should have been on the order of ~0.02 pH units, due to the relatively small seasonal variability in pH_sw_ at Rottnest Island (~0.07; Supplementary Fig. S2). In contrast, seasonal variations in pH_cf_ are an order of magnitude higher (>0.2 pH units). Therefore, our results unequivocally demonstrate that seawater pH is not the major factor driving seasonal changes in coral pH_cf_ up-regulation in this study.

Our current understanding of the sensitivity of the calcifying fluid chemical composition to changes in seawater carbonate chemistry has generally been inferred from controlled laboratory experiments^14,47–51^, which have kept other environmental conditions constant, such as temperature^14,47–51^ and/or light^14,50,51^. However, our findings demonstrate that other environmental factors beyond pH_sw_ may have a much larger influence on pH_cf_ and [CO_3_^2−^]_cf_, at least on seasonal time-scales. For instance, the observed inverse relationship between seasonally varying pH_cf_ and DIC_cf_ is suggestive of a deliberate mechanism to compensate for seasonal declines in temperature and light, and thus changes to the supply and/or transport of DIC necessary for coral growth^21^. The lower levels of DIC in the calcifying fluid during winter reflect a reduction in rates of carbon fixation by endosymbionts in winter and are compensated for by higher pH_cf_ up-regulation (Fig. 3). Despite the stabilizing effects that a counter-cyclical seasonal relationship between pH_cf_ and DIC_cf_ would have on Ω_cf_, we nonetheless show that Ω_cf_ also varies seasonally. This seasonal behaviour of Ω_cf_ may, at the very least, dampen any expected seasonality in rates of coral calcification.

To better constrain the relative sensitivity of coral calcification rates to seasonal changes in both temperature and the carbonate chemistry of the calcifying fluid, calcification rates were modelled using the inorganic rate equation (see Eq. 5 in Methods)^12^. In this model, aragonite precipitation occurs according to abiotic rate kinetics under chemical conditions dictated by the living coral (e.g., elevated pH_cf_ and DIC_cf_). Three scenarios are considered: (1) temperature and Ω_cf_ vary with season, (2) temperature, DIC_cf_ and Ω_cf_ vary with season while pH_cf_ is calculated based on seawater pH_sw_ in accordance with the results of fixed aquaria experiments^40,49^, and (3) Ω_cf_ varies with season, but temperature is kept constant at its annual average (21.7 °C). Modelled rates of calcification based on inorganic rate kinetics are a factor of 2 to 7 times lower than the measured calcification rates (see Supplementary Table S4). This indicates that an estimated Ω_cf_ of 30 to 50 is required to attain the measured rates of calcification and thus much higher than our seasonal range of ~10 to 16, depending on taxa (Fig. 3d). Thus, physiological mechanisms must be operative in enhancing rates of skeletal precipitation relative to that expected from inorganic rate kinetics.

**Figure 5:**
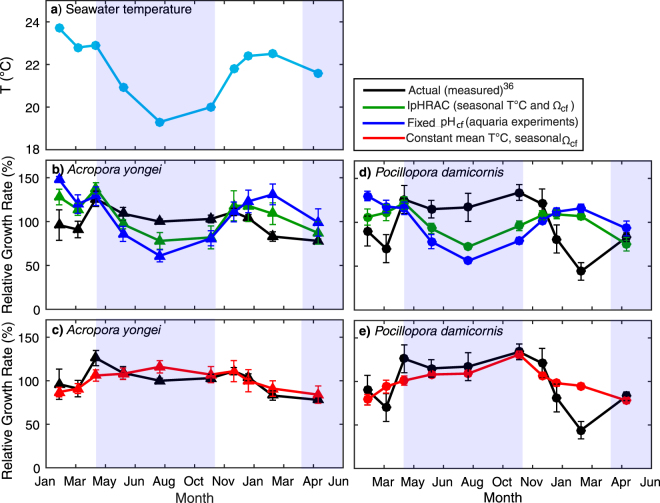
Measured and modelled seasonal growth rate response for branching *Acropora yongei* and *Pocillopora damicornis at* Rottnest Island. (**a**) Seasonal changes in average seawater temperature (°C). Actual calcification rates measured using the buoyant weight technique^36^ and predicted calcification rates modelled using inorganic rate kinetics for (**b,c**) *A. yongei*, and (**d,e**) *P. damicornis*. Black symbols represent the measured calcification rates (mean ± 1 SE) for *A. yongei* (*n* = 16) and *P. damicornis* (*n* = 9)^36^. Green symbols represent the predicted calcification rates using seasonally varying temperature and seasonally varying Ω_cf_, blue symbols represent the predicted calcification rates using pH_cf_ calculated from fixed condition experiments for *Acropora* spp., (y = 0.51pH_sw_ + 4.28^40,49^; where pH_sw_ ranged from 8.03–8.10), and red symbols represent the predicted rates using a constant mean temperature (21.7 °C) and seasonally varying Ω_cf._ All growth rates are expressed as percentage relative to the mean. Light blue shading denotes winter and unshaded areas denote summer, defined based on seasonal changes in temperature and light.

During summer, the higher DIC_cf_ supply is offset by a systematic reduction in pH_cf_ to modulate Ω_cf_ and calcification rates. While the estimated calcification rates from inorganic rate kinetics in scenario 1 are still ~50% higher at the peak in summer compared to the minima in winter, this seasonal change is nevertheless substantially less than the estimated ~90% seasonal change in calcification rates if pH_cf_ levels were more or less constant year-round, as inferred from fixed condition aquaria experiments. Calcification rates estimated from both scenarios indicate that the seasonal variation in the measured coral calcification rates should be pro-cyclical with temperature and much greater than observed^36^ (Fig. 5). Thus, scenarios 1 and 2, which allow for the full seasonal variation in temperature to be expressed through the inorganic rate kinetics, exhibit large deviations from the measured calcification rates for *A. yongei* (Scenario 1: RMSE = 17.0%; Scenario 2: RMSE = 30.3%), and *P. damicornis* (Scenario 1: RMSE = 32.7%; Scenario 2: RMSE = 43.3%). There is a strong physiological control over Ω_cf_ such that calcification rates appear to be modulated more by Ω_cf_, rather than temperature directly. We find that the calcification rates modelled under a constant mean temperature (i.e., scenario 3) show the best fit to the observed seasonal patterns in calcification (RMSE = 11.5% and 20.8%, respectively; Fig. 5). One possibility is that differences between the complex skeletal micro-architecture of coral growth (e.g., a higher active surface area on which precipitation can occur)^15^ compared to abiotic aragonite crystal formation could result in the enhanced rates of biologically-mediated coral calcification shown here. Another possibility is that other factors may influence seasonal patterns in rates of calcification, for example, changes in the speed at which the corals create organic matrices and crystal templates^52–56^ necessary for mineralization. This cannot be ruled out given that the calcification rate measurements are integrated over several weeks of biomineralization.

Although the relatively seasonally invariant calcification rates for corals from Rottnest Island stand in contrast with findings from more tropical environments^24,33–35^, there are also a number of studies^36,39,57,58^ that show limited seasonal variability in calcification rates for several coral species. These studies were conducted across a range of locations (i.e., spanning 10 degrees latitude) in Western Australia, and are thus widely applicable to reef-building corals growing in a various environments. There have been a number of hypotheses to explain this lack of seasonality, ranging from the residual effects of sub-lethal thermal stress following an unusually strong marine heat wave^39^, to higher rates of particulate nutrient uptake in winter^36,37,57^. In the present study, however, the limited seasonal variability in calcification rates can be explained by the seasonally counter-cyclical up-regulation of pH_cf_ and DIC_cf_ to deliberately elevate Ω_cf_ and support near-constant rates of calcification year-round, despite much cooler temperatures during winter. This apparent physiological control over the internal carbonate chemistry is further supported by the results from the Papua New Guinea CO_2_ seeps^16^ and Free Ocean Carbon Enrichment experiment (FOCE)^11^; both of which demonstrated the mechanism of pH_cf_ ‘homeostasis’ in corals despite exposure to extreme variations in ambient pH_sw_.

We have now identified a key mechanism of chemical regulation within the calcifying fluid composition that assists reef-building coral to calcify year-round in sub-tropical conditions (i.e., lower and more seasonally variable temperature and light). While these findings are based on branching corals at Rottnest Island, they nevertheless demonstrate a key physiological mechanism whereby changes in the calcifying fluid carbonate chemistry act to modulate calcification rates. Although such robust regulation of chemical conditions during calcification will help protect the growth of adult corals from the influence of declining seawater pH^12,15^, it will not necessarily preclude corals from being vulnerable to thermal stress^59^. Thus, the future survival of coral reefs in the face of Earth’s rapidly changing climate will ultimately depend on the capacity of reef-building coral to endure the increasingly frequent and intense CO_2_-driven warming events^59^.

## Methods

### Overview

This study was conducted at Salmon Bay on the south side of Rottnest Island, which is located approximately 20 km west of Perth, WA (32°S, 115°E). See Ross *et al*.^36^ for a detailed map of study area. Single individual branches of *A. yongei* and *P. damicornis* were collected from four naturally growing colonies (1 branch per colony) every 1 to 3 months between February 2013 and March 2015 and subject to the geochemical analyses described herein.

### Environmental data

Continuous measurements of seawater pH_T_ (Total scale, with ±0.03 accuracy) were made using a SeaFET ocean pH sensor (Satlantic, Canada) in October 2014 (spring) and April 2015 (autumn). Earlier measurements were made in July 2013 (winter) and February 2014 (summer)^36^. Seawater temperature was continuously measured at the study site for the entire duration of the study using HOBO temperature loggers (±0.2 °C, Onset Computer Corp.). Daily down-welling planar photo-synthetically active radiation (PAR in mol m^−2^ d^−1^) was measured from December 2013 to July of 2014 using Odyssey light loggers (±5%, Odyssey Data Recording) that had been calibrated against a high-precision LiCor 192 A cosine PAR (±5%, LiCor Scientific) sensor^36^. For this location, seasons were defined as follows: winter from mid-April through to mid-October and summer from mid-November through to mid-April.

### Boron isotopic and trace element analyses

The δ^11^B of coral skeletons were measured from the uppermost apical section of the growing tip of *A. yongei* and *P. damicornis* skeletons (Supplementary Fig. S4, Supplementary Table S5). We sampled ~4 mm of the apical growing tip based on our previous study^36^ showing average monthly extension rates of ~50 mm yr^−1^ (or ~4 mm month^−1^). Alternate methods for determining the sclero-chronology of the deposited skeletal material include using fluorescent staining^60^ or isotope labelling, which are informative for analysis by laser ablation-multi-collector-ICP-MS^61^. Unfortunately, repeated labelling of the coral colonies used in this study was not feasible due to the frequent (1 to 3 month) skeletal sampling resolution and long study period (i.e., ~2 years). Instead, we used molar ratios of strontium to calcium (Sr/Ca) and lithium to magnesium (Li/Mg) in the most recently deposited skeletal material and well-known, highly correlated relationships between these trace element ratios and ambient seawater temperature^62^ to confirm the seasonal chronology of skeletal growth histories^11^.

Powders derived from the temporally controlled samples of coral skeleton were cleaned^63^ and dissolved in 0.58 N HNO_3_. Aliquots of these acidified samples were analysed for trace elements (Sr/Ca, Li/Mg, and B/Ca) using an X-Series 2 Quadrupole Inductively Coupled Plasma Mass Spectrometer (Thermo Fisher Scientific). The extraction and concentration of boron-rich solutions from acidified sample solutions was performed via paired cation-anion resin columns and analysed with a NU Plasma II (Nu Instruments, Wrexham, UK) multi-collector inductively coupled plasma mass spectrometer (MC-ICPMS)^64^.

### Boron isotope pH-proxy

We determined the pH of the calcifying fluid from the measured δ^11^B values according to the following equation^65^:

1 $$p{H}_{cf}=\frac{p{K}_{B}-Log\{[{\delta }^{11}{B}_{sw}-{\delta }^{11}{B}_{carb}]}{[{\alpha }_{B}{\delta }^{11}{B}_{carb}-\,{\delta }^{11}{B}_{sw}+1000(1.0272-1]\}}$$

where *pK*_B_ is the dissociation constant of boric acid in seawater^66^ at the temperature and salinity of the seawater in Salmon Bay, δ^11^B_carb_ and δ^11^B_SW_ are the boron isotopic composition of the coral skeleton and average seawater, respectively, and α_B_ is the isotopic fractionation factor (1.0272)^67^.

### Estimation of DIC_cf_ and modelled rates of mineral precipitation

We estimate the concentration of carbonate ions at the site of calcification ([CO_3_^2−^]_cf_) using molar ratios of boron to calcium (B/Ca) according to the following relationship^45^ simplified by McCulloch, *et al*.^21^:

2 $${[C{O}_{3}^{2-}]}_{cf}=\frac{{[B{(OH)}_{4}^{-}]}_{cf}{K}_{D}^{B/Ca}}{{[B/Ca]}_{arag}}$$

where [B(OH)_4_^–^]_cf_ is the concentration of borate in the calcifying fluid, ${K}_{D}^{B/Ca}$ is the molar distribution coefficient for boron in aragonite, and [B/Ca]_arag_ is the elemental ratio of boron to calcium in the coral skeleton. To estimate [B(OH)_4_^–^]_cf_, we assume that the concentration of total inorganic boron in the calcifying fluid is salinity dependent^68,69^, and equal to that of the surrounding seawater. The strong linear relationship between the calculated [CO_3_^2−^]_cf_(using eq. 2) and the measured [CO_3_^2−^]_cf_ from abiogenic experiments by Holcomb, *et al.* ^45^ is shown in Supplementary Fig. S5. The relative amounts of borate are determined by the pH_cf_^66^, as determined from the δ^11^B isotopic measurements (see eq. 1). The ${K}_{D}^{B/Ca}$ is calculated as a function of pH_cf_ according to^21,45^:

3 $${K}_{D}^{B/Ca}=0.00297\exp (-0.0202\,\,{[{H}^{+}]}_{cf})$$

where [H^+^] in the calcifying fluid is estimated from the coral δ^11^B derived pH_cf_ and only varies by less than ± 3% over the range in which most coral pH_cf_ are known to occur (8.3 to 8.6)^21^.

The concentration of DIC_cf_ is then calculated from pH_cf_ (eq. 1) and [CO_3_^2−^]_cf_ (eq. 2).

The Ω_cf_ is estimated from [Ca^2+^] and [CO_3_^2−^] (from δ^11^B and B/Ca) according to the following relationship:

4 $${{\rm{\Omega }}}_{cf}=\,\frac{[C{a}^{2+}][C{O}_{3}^{2-}]}{{K}_{sp}^{\ast }}$$

where *K* ^*^_sp_ is the solubility constant for aragonite as a function of temperature and salinity and [Ca^2+^] is assumed to be the same as the surrounding seawater values.

Finally, we used the combination of Ω_cf_ and temperature to infer rates of abiotic aragonite precipitation (G) at the site of calcification according to the model of internal pH-regulation abiotic calcification model (IpHRAC)^12^:

5 $$G=k{({{\rm{\Omega }}}_{cf}-1)}^{n}$$

where *k* and *n* are temperature-dependent empirical constants^25^.

### Observed rates of coral calcification

Calcification rates (mg CaCO_3_ cm^−2^ d^−1^) for both *A. yongei* (*n* = 16) and *P. damicornis* (*n* = 9) were measured^36^ on individual coral colonies (mounted on plastic tiles and deployed *in-situ*) using the buoyant weight technique^70,71^. These calcification measurements were conducted on colonies from the same location and at the same time as when branches were collected (from separate colonies) for the analysis of skeletal geochemistry. This field-based approach allowed the comparison of seasonal changes of *in-situ* determined coral calcification rates^36^ with concomitant changes in seawater temperature, pH and DIC together with coral calcifying fluid pH_cf_ and DIC_cf_.

### Availability of data

Data is available at the Zenodo Digital Repository (DOI: 10.5281/zenodo.1009710).

## Electronic supplementary material

Supplementary Material
