# Supplementary material for: Active modulation of the calcifying fluid carbonate chemistry (δ11B, B/Ca) and seasonally invariant coral calcification at sub-tropical limits
Source: Sci Rep. 2017 Oct 23;7:13830. doi: 10.1038/s41598-017-14066-9 (PMC5653831; doi:10.1038/s41598-017-14066-9)
Supplement: Supplementary file 1 — Supplementary Material [file 41598_2017_14066_MOESM1_ESM.pdf]

Supplementary information for "Active modulation of the calcifying fluid carbonate chemistry (δ^{11}B, B/Ca) and seasonally invariant coral calcification at sub-tropical limits"

Claire L. Ross[1, 2*], James L. Falter[1, 2], Malcolm T. McCulloch[1, 2]

[1]Oceans Institute and School of Earth Sciences, The University of Western Australia
[2]Australian Research Council Centre of Excellence for Coral Reef Studies, The University of Western Australia

*Corresponding author: Claire Ross (claire.ross@research.uwa.edu.au)

Contents of this file

Supplementary figures 1–5

Supplementary Tables 1–5

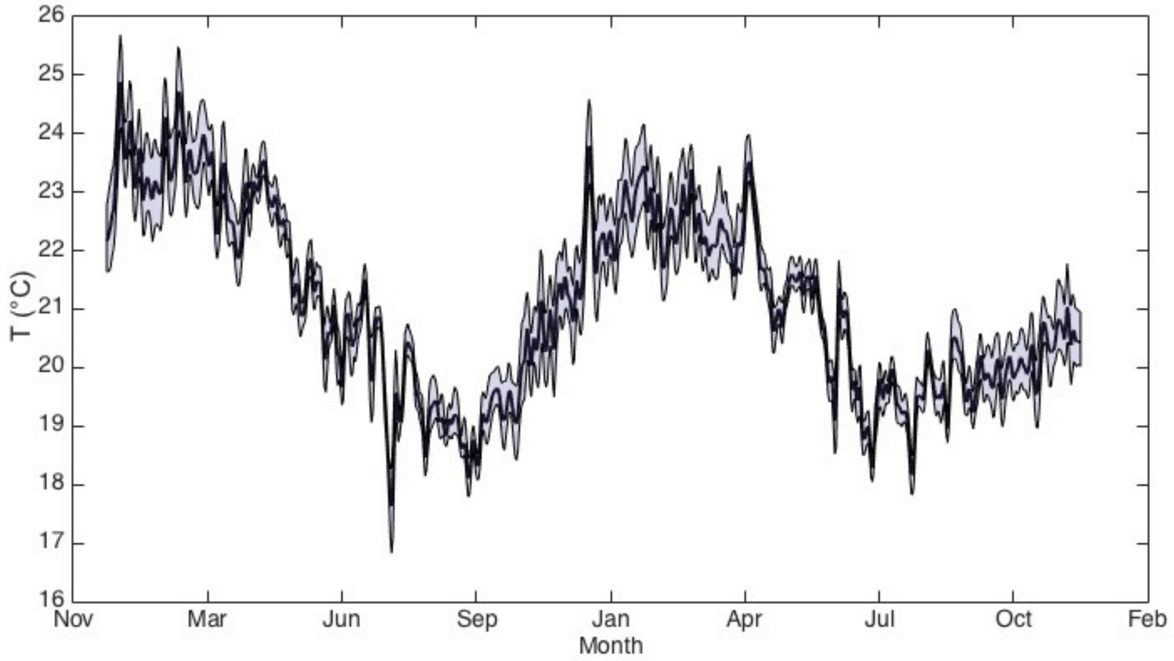

Supplementary Figure S1. Seasonal changes in seawater temperature at Rottnest Island. Daily averaged water temperature at Salmon Bay, Rottnest Island from December 2012 through December 2014. The heavy black line shows the average daily temperatures while the grey regions represent the range between hourly minimum and maximum temperatures for each day.

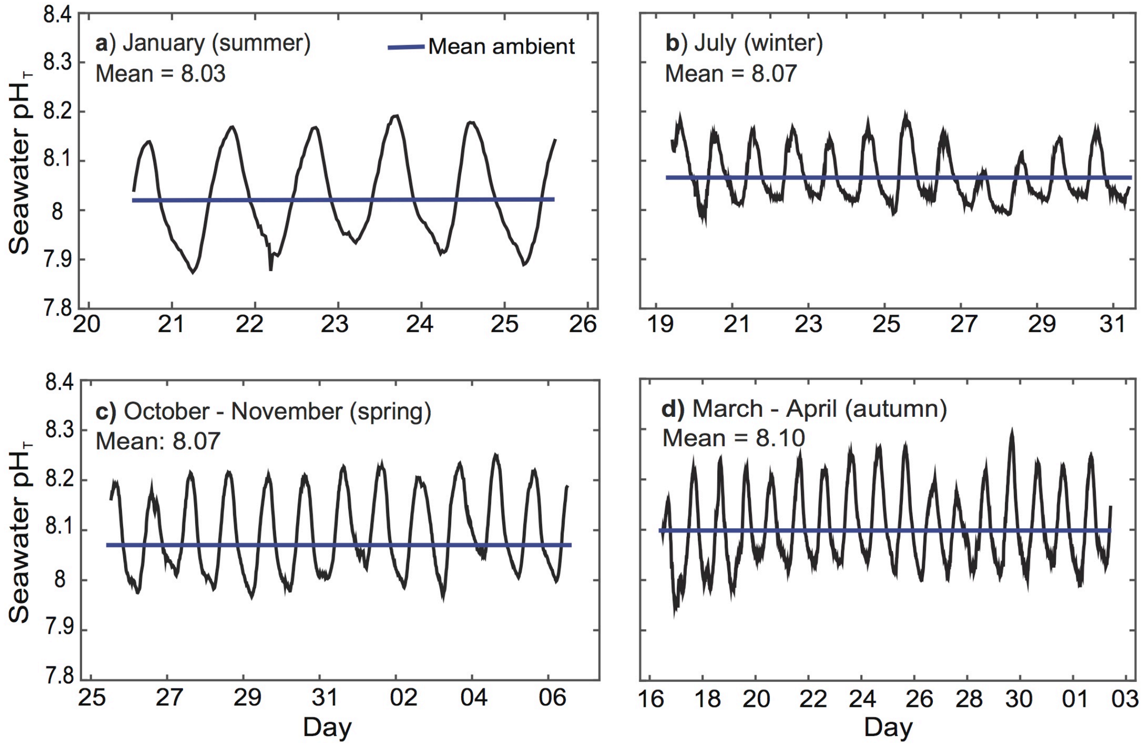

Supplementary Figure S2. Seasonal seawater pH_T at Rottnest Island. Diurnal variations of seawater pH_T at Salmon Bay, Rottnest Island taken during (**a**) 20th–25th of January 2014[36], (**b**) 19th–30th of July 2014, (**c**) 25th Oct–6th Nov 2014 and (**d**) 16th Mar–1st April 2015. The solid blue lines denote the average pH_T over the period of deployment.

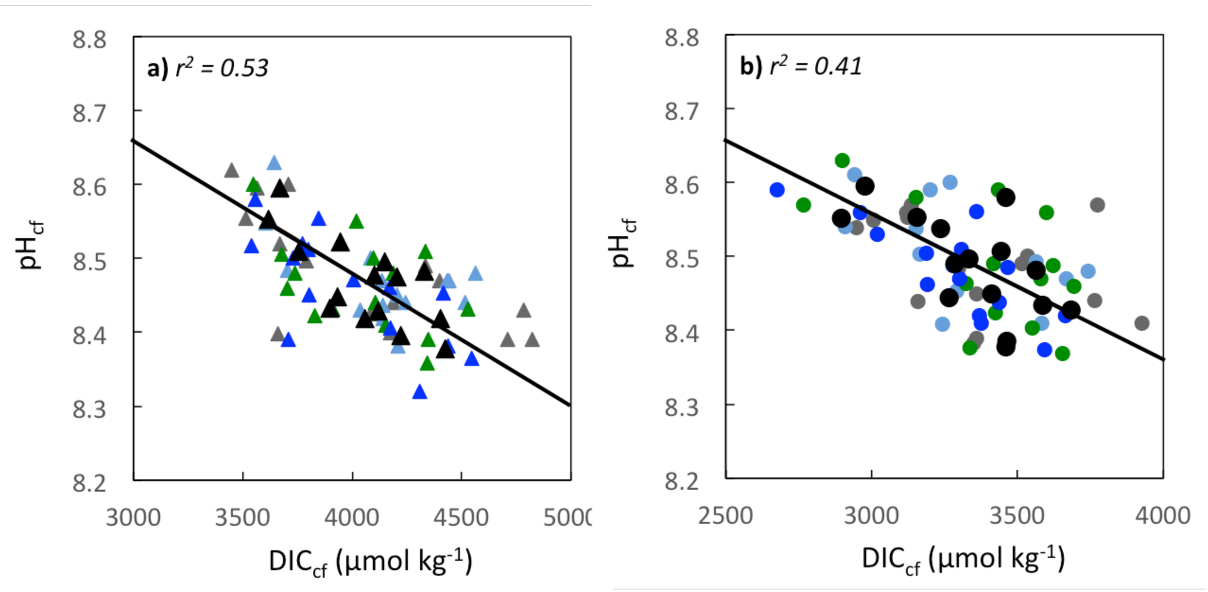

Supplementary Figure S3. Inverse relationships between seasonal pH$_{cf}$ versus DIC$_{cf}$. Coral pH$_{cf}$ plotted against DIC$_{cf}$ for (**a**) *Acropora yongei* (pH$_{cf}$ = -0.0002 DIC$_{cf}$ + 9.17) and (**b**) *Pocillopora damicornis* (pH$_{cf}$ = -0.0002 DIC$_{cf}$ + 9.21). Coloured symbols represent each colony while the black symbols denote the mean ± 1 SE (n = 4; colony 1 = grey, colony 2 = light blue, colony 3 = green, colony 4 = dark blue) for each time point.

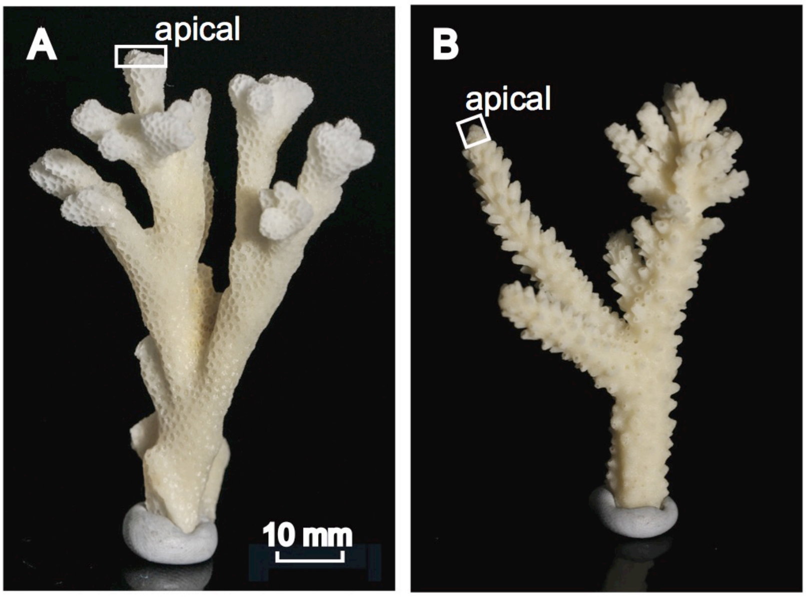

Supplementary Figure S4. Region sampled for geochemical measurements. Sampling schematic showing the apical location sampled for (**a**) *Pocillopora damicornis*, and (**b**) *Acropora yongei* (photographs taken by C. Ross).

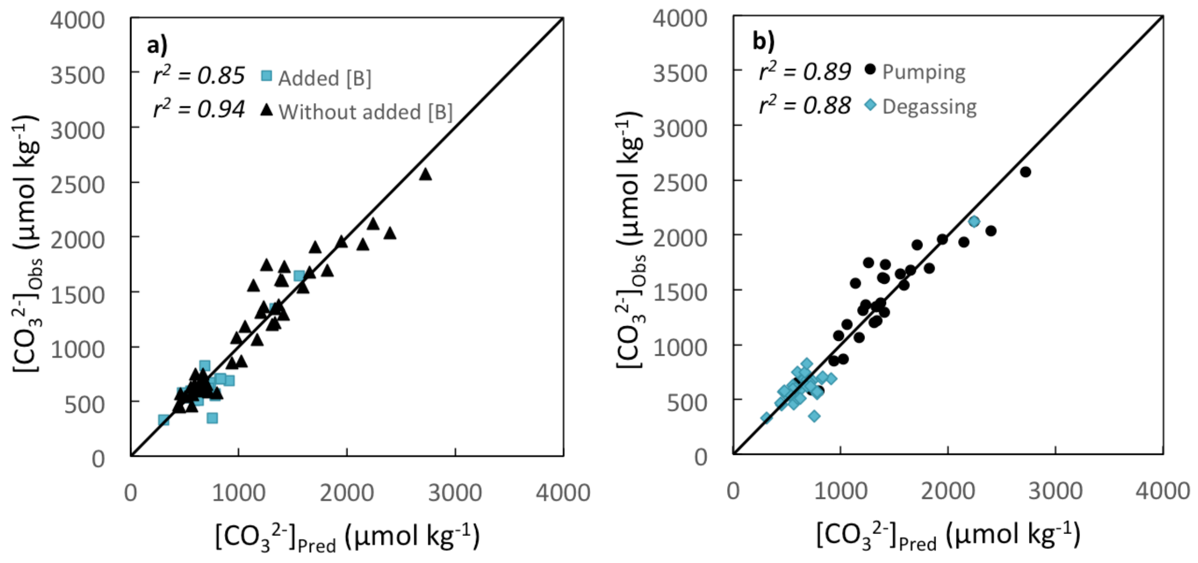

Supplementary Figure S5. Relationships between predicted $[CO_3^{2-}]_{pred}$ (using Eq. 2)[21] and measured $[CO_3^{2-}]_{obs}$ from inorganic experiments[45]. Symbols are the means of different experiments; (**a**) with added B and without added B, and (**b**) pumping experiments and degassing experiments. Both plots show 1:1 regression line. The relationship between the predicted and observed[45] for all data combined is $[CO_3^{2-}]_{obs} = 0.97[CO_3^{2-}]_{pred} + 19.87$ (mean absolute error of ± 116 µmol kg^{-1}; $r^2 = 0.93$, $n = 64$).

Supplementary Tables

Supplementary Table S1. Seasonal changes in environmental conditions at the study site. Environmental data (mean ± 1 SE) measured at Rottnest Island, Western Australia[36].

Variable	Units	Summer	Winter
Temperature	°C	23.0 ± 0.2	19.5 ± 0.2
Light	mol m^{-2} d^{-1}	45.9 ± 0.6	19.3 ± 0.3
Total Alkalinity	µmol kg^{-1}	2,282 ± 18	2,297 ± 4
pCO_2	µatm	310 ± 20	339 ± 17
Carbonate	µmol kg^{-1}	248 ± 9	211 ± 8
DIC	µmol kg^{-1}	1928 ± 20	1997 ± 14
Ω_{sw}	-	3.89 ± 0.14	3.28 ± 0.12
Ammonium	mmol m^{-3}	0.50 ± 0.03	0.43 ± 0.06
Nitrate	mmol m^{-3}	0.25 ± 0.04	0.77 ± 0.08
Phosphate	mmol m^{-3}	0.03 ± 0.01	0.19 ± 0.04

Supplementary Table S2. Coral skeletal B/Ca, Li/Mg and Sr/Ca ratios, and $\delta^{11}B$ values.

The measurements of skeletal B/Ca, Li/Mg and Sr/Ca ratios and $\delta^{11}B$ values for *Acropora yongei* and *Pocillopora damicornis* (mean ± 1 SE) at Rottnest Island, Western Australia.

Date	***A. yongei***							
	**B/Ca**	**SE**	**Li/Mg**	**SE**	**Sr/Ca**	**SE**	**$\delta^{11}B$**	**SE**
	mmol mol^{-1}		mmol mol^{-1}		mmol mol^{-1}		‰	
Feb-13	0.51	0.07	1.84	0.01	9.46	0.03	22.36	0.40
Mar-13	0.54	0.19	1.90	0.01	9.58	0.02	23.04	0.41
Apr-13	0.52	0.28	1.95	0.02	9.57	0.01	23.35	0.17
Jun-13	0.54	0.25	1.97	0.03	9.68	0.03	23.79	0.42
Aug-13	0.58	0.16	2.12	0.03	9.79	0.04	25.18	0.29
Oct-13	0.52	0.06	2.04	0.03	9.63	0.03	22.85	0.06
Dec-13	0.50	0.23	1.81	0.02	9.51	0.02	23.35	0.31
Jan-14	0.49	0.26	1.80	0.03	9.50	0.03	22.58	0.38
Feb-14	0.49	0.12	1.85	0.02	9.63	0.01	21.98	0.16
Apr-14	0.56	0.18	1.96	0.02	9.58	0.03	22.76	0.18
Jul-14	0.59	0.08	2.05	0.06	9.76	0.05	24.08	0.25
Aug-14	0.58	0.06	2.20	0.02	9.69	0.01	23.37	0.22
Oct-14	0.53	0.23	2.08	0.02	9.74	0.04	23.16	0.55
Dec-14	0.54	0.11	2.05	0.02	9.66	0.01	22.16	0.06
Mar-15	0.52	0.03	1.92	0.03	9.56	0.02	22.57	0.29
**Date**	***P. damicornis***							
	**B/Ca**	**SE**	**Li/Mg**	**SE**	**Sr/Ca**	**SE**	**$\delta^{11}B$**	**SE**
	mmol mol^{-1}		mmol mol^{-1}		mmol mol^{-1}		‰	
Feb-13	0.62	0.14	1.71	0.05	9.30	0.02	22.24	0.54
Mar-13	0.62	0.09	1.81	0.02	9.36	0.02	23.05	0.45
Apr-13	0.63	0.14	1.84	0.03	9.36	0.03	23.48	0.62
Jun-13	0.66	0.22	1.92	0.07	9.45	0.06	24.25	0.81
Aug-13	0.71	0.28	1.90	0.02	9.47	0.04	24.76	0.40
Oct-13	0.61	0.20	1.87	0.04	9.53	0.02	24.41	0.31
Dec-13	0.60	0.17	1.85	0.02	9.45	0.03	23.35	0.25
Jan-14	0.59	0.25	1.84	0.01	9.39	0.02	22.79	0.60
Feb-14	0.60	0.16	1.75	0.03	9.36	0.03	22.98	0.42
Apr-14	0.63	0.17	1.81	0.04	9.43	0.02	22.11	0.26
Jul-14	0.66	0.25	2.02	0.03	9.58	0.04	23.78	0.39
Aug-14	0.74	0.11	1.96	0.03	9.54	0.02	24.01	0.24
Oct-14	0.63	0.18	2.01	0.03	9.58	0.02	23.31	0.59
Dec-14	0.66	0.07	1.90	0.02	9.50	0.03	23.24	0.42
Mar-15	0.66	0.11	1.85	0.02	9.42	0.01	22.86	0.32

Supplementary Table S3. Coral carbonate ion concentrations $[CO_3^{2-}]_{cf}$ within the calcifying fluid. The $[CO_3^{2-}]_{cf}$ estimated from boron to calcium ratios (B/Ca) for *Acropora yongei* and *Pocillopora damicornis* (mean ± 1 SE, *n* = 4) at Rottnest Island, Western Australia.

	A. yongei		***P. damiconris***	
**Date**	**$[CO_3^{2-}]_{cf}$**	**SE**	**$[CO_3^{2-}]_{cf}$**	**SE**
Feb-13	874.0	35.1	694.5	33.8
Mar-13	872.8	44.6	755.6	6.8
Apr-13	959.8	52.4	804.5	15.6
Jun-13	938.1	35.9	791.2	22.1
Aug-13	946.9	22.0	769.8	29.4
Oct-13	898.3	19.2	888.4	25.3
Dec-13	989.0	24.5	815.5	20.0
Jan-14	911.3	31.1	777.7	28.9
Feb-14	856.4	27.7	769.1	34.4
Apr-14	810.9	37.9	658.1	7.5
Jul-14	878.4	19.5	767.0	40.8
Aug-14	839.9	29.3	697.5	8.7
Oct-14	903.3	9.0	769.7	10.1
Dec-14	802.3	15.2	738.3	15.6
Mar-15	876.0	18.0	710.1	9.1

Supplementary Table S4. Seasonal changes in measured and modelled calcification rates for branching *Acropora yongei* and *Pocillopora damicornis at* Rottnest Island. Measured mean calcification rates[1] (mg cm^{-2} d^{-1})[36] and predicted mean absolute calcification rates (mg cm^{-2} d^{-1}) using the three model scenarios. Scenario 1 are calcification rates modelled using inorganic rate kinetics using seasonally varying temperature and Ω_{cf}, scenario 2 are the rates estimated using the pH_{cf} values calculated from fixed condition experiments for *Acropora* spp., ($y = 0.51pH_{sw} + 4.28$[40,49]; pH_{sw} ranged from 8.03–8.10), and scenario 3 are the estimated relative rates using a constant mean temperature (21.7°C) and seasonally varying $\Omega_{cf.}$ Light blue shading denotes winter and unshaded areas denote summer.

	**Date**	**Growth Rates (mg cm^{-2} d^{-1})**			
		**Measured**	**IpHRAC**	**pH_{cf} (fixed aquaria experiments)**	**Constant T°C, Variable Ω_{cf}**
Acropora yongei	Feb-13	1.54	0.54	0.54	0.36
	Mar-13	1.46	0.49	0.44	0.38
	Apr-13	2.02	0.57	0.47	0.43
	Jun-13	1.76	0.42	0.32	0.46
	Aug-13	1.61	0.34	0.22	0.50
	Oct-13	1.66	0.35	0.30	0.45
	Dec-13	1.79	0.50	0.41	0.46
	Jan-14	1.67	0.50	0.45	0.41
	Feb-14	1.33	0.45	0.48	0.37
	Apr-14	1.25	0.40	0.36	0.39
Pocillopora damicornis	Feb-13	0.60	0.36	0.39	0.25
	Mar-13	0.47	0.39	0.35	0.31
	Apr-13	0.84	0.43	0.34	0.33
	Jun-13	0.77	0.33	0.23	0.36
	Aug-13	0.79	0.25	0.17	0.36
	Oct-13	0.90	0.34	0.23	0.44
	Dec-13	0.81	0.38	0.30	0.35
	Jan-14	0.54	0.38	0.33	0.32
	Feb-14	0.30	0.37	0.35	0.31
	Apr-14	0.55	0.26	0.28	0.25

Supplementary Table S5. Coral skeletal $\delta^{11}B$ values (± 2 SD). The individual measurements of skeletal $\delta^{11}B$ for *Acropora yongei* and *Pocillopora damicornis.*

Location / Species	Date Collected	$d^{11}B_{(carb)}$ (permil)	Error (± 2s int)
Rottnest Island - *Acropora yongei*	03-Mar-13	23.02	0.04
Rottnest Island - *Acropora yongei*	03-Mar-13	22.83	0.05
Rottnest Island - *Acropora yongei*	03-Mar-13	22.35	0.04
Rottnest Island - *Acropora yongei*	03-Mar-13	21.25	0.07
Rottnest Island - *Acropora yongei*	05-Apr-13	24.14	0.05
Rottnest Island - *Acropora yongei*	05-Apr-13	22.88	0.04
Rottnest Island - *Acropora yongei*	05-Apr-13	22.96	0.05
Rottnest Island - *Acropora yongei*	05-Apr-13	22.18	0.06
Rottnest Island - *Acropora yongei*	06-May-13	23.02	0.05
Rottnest Island - *Acropora yongei*	06-May-13	23.30	0.05
Rottnest Island - *Acropora yongei*	06-May-13	23.24	0.05
Rottnest Island - *Acropora yongei*	06-May-13	23.84	0.06
Rottnest Island - *Acropora yongei*	20-Jun-13	25.00	0.04
Rottnest Island - *Acropora yongei*	20-Jun-13	23.04	0.06
Rottnest Island - *Acropora yongei*	20-Jun-13	23.46	0.04
Rottnest Island - *Acropora yongei*	20-Jun-13	23.68	0.10
Rottnest Island - *Acropora yongei*	21-Aug-13	25.18	0.04
Rottnest Island - *Acropora yongei*	21-Aug-13	25.28	0.04
Rottnest Island - *Acropora yongei*	21-Aug-13	24.06	0.04
Rottnest Island - *Acropora yongei*	21-Aug-13	26.20	0.06
Rottnest Island - *Acropora yongei*	15-Nov-13	22.97	0.05
Rottnest Island - *Acropora yongei*	15-Nov-13	22.74	0.04
Rottnest Island - *Acropora yongei*	15-Nov-13	22.96	0.06
Rottnest Island - *Acropora yongei*	15-Nov-13	22.74	0.10
Rottnest Island - *Acropora yongei*	23-Dec-13	22.54	0.04
Rottnest Island - *Acropora yongei*	23-Dec-13	23.21	0.05
Rottnest Island - *Acropora yongei*	23-Dec-13	23.77	0.04
Rottnest Island - *Acropora yongei*	23-Dec-13	23.90	0.06
Rottnest Island - *Acropora yongei*	17-Jan-14	22.22	0.05
Rottnest Island - *Acropora yongei*	17-Jan-14	23.04	0.04
Rottnest Island - *Acropora yongei*	17-Jan-14	23.37	0.06
Rottnest Island - *Acropora yongei*	17-Jan-14	21.69	0.11
Rottnest Island - *Acropora yongei*	27-Feb-14	22.38	0.05
Rottnest Island - *Acropora yongei*	27-Feb-14	21.96	0.06
Rottnest Island - *Acropora yongei*	27-Feb-14	21.62	0.06
Rottnest Island - *Acropora yongei*	27-Feb-14	21.96	0.06
Rottnest Island - *Acropora yongei*	14-May-14	22.25	0.06
Rottnest Island - *Acropora yongei*	14-May-14	23.06	0.04
Rottnest Island - *Acropora yongei*	14-May-14	22.74	0.06
Rottnest Island - *Acropora yongei*	14-May-14	22.98	0.03
Rottnest Island - *Acropora yongei*	18-Jul-14	24.12	0.06
Rottnest Island - *Acropora yongei*	18-Jul-14	23.93	0.06
Rottnest Island - *Acropora yongei*	18-Jul-14	24.73	0.13
Rottnest Island - *Acropora yongei*	18-Jul-14	23.54	0.05

Rottnest Island - *Acropora yongei*	02-Sep-14	23.11	0.06
Rottnest Island - *Acropora yongei*	02-Sep-14	22.99	0.06
Rottnest Island - *Acropora yongei*	02-Sep-14	23.38	0.07
Rottnest Island - *Acropora yongei*	02-Sep-14	23.98	0.12
Rottnest Island - *Acropora yongei*	24-Oct-14	24.69	0.06
Rottnest Island - *Acropora yongei*	24-Oct-14	23.22	0.05
Rottnest Island - *Acropora yongei*	24-Oct-14	22.20	0.04
Rottnest Island - *Acropora yongei*	24-Oct-14	22.52	0.06
Rottnest Island - *Acropora yongei*	19-Dec-14	22.27	0.05
Rottnest Island - *Acropora yongei*	19-Dec-14	22.17	0.07
Rottnest Island - *Acropora yongei*	19-Dec-14	22.23	0.07
Rottnest Island - *Acropora yongei*	19-Dec-14	21.99	0.06
Rottnest Island - *Acropora yongei*	31-Mar-15	21.91	0.12
Rottnest Island - *Acropora yongei*	31-Mar-15	22.64	0.16
Rottnest Island - *Acropora yongei*	31-Mar-15	22.45	0.14
Rottnest Island - *Acropora yongei*	31-Mar-15	23.29	0.15
Rottnest Island - *Pocillopora damicornis*	03-Mar-13	22.31	0.04
Rottnest Island - *Pocillopora damicornis*	03-Mar-13	21.56	0.05
Rottnest Island - *Pocillopora damicornis*	03-Mar-13	22.23	0.05
Rottnest Island - *Pocillopora damicornis*	03-Mar-13	22.87	0.04
Rottnest Island - *Pocillopora damicornis*	05-Apr-13	23.56	0.04
Rottnest Island - *Pocillopora damicornis*	05-Apr-13	22.51	0.07
Rottnest Island - *Pocillopora damicornis*	05-Apr-13	23.25	0.05
Rottnest Island - *Pocillopora damicornis*	05-Apr-13	22.87	0.06
Rottnest Island - *Pocillopora damicornis*	06-May-13	24.36	0.05
Rottnest Island - *Pocillopora damicornis*	06-May-13	23.45	0.04
Rottnest Island - *Pocillopora damicornis*	06-May-13	23.07	0.04
Rottnest Island - *Pocillopora damicornis*	06-May-13	23.04	0.06
Rottnest Island - *Pocillopora damicornis*	20-Jun-13	23.27	0.04
Rottnest Island - *Pocillopora damicornis*	20-Jun-13	25.08	0.05
Rottnest Island - *Pocillopora damicornis*	20-Jun-13	24.73	0.04
Rottnest Island - *Pocillopora damicornis*	20-Jun-13	23.94	0.06
Rottnest Island - *Pocillopora damicornis*	21-Aug-13	24.40	0.04
Rottnest Island - *Pocillopora damicornis*	21-Aug-13	24.59	0.04
Rottnest Island - *Pocillopora damicornis*	21-Aug-13	25.33	0.04
Rottnest Island - *Pocillopora damicornis*	21-Aug-13	24.72	0.07
Rottnest Island - *Pocillopora damicornis*	15-Nov-13	24.19	0.05
Rottnest Island - *Pocillopora damicornis*	15-Nov-13	24.75	0.05
Rottnest Island - *Pocillopora damicornis*	15-Nov-13	24.60	0.04
Rottnest Island - *Pocillopora damicornis*	15-Nov-13	24.11	0.06
Rottnest Island - *Pocillopora damicornis*	23-Dec-13	23.57	0.04
Rottnest Island - *Pocillopora damicornis*	23-Dec-13	23.39	0.04
Rottnest Island - *Pocillopora damicornis*	23-Dec-13	22.99	0.04
Rottnest Island - *Pocillopora damicornis*	23-Dec-13	23.44	0.07
Rottnest Island - *Pocillopora damicornis*	17-Jan-14	22.53	0.04
Rottnest Island - *Pocillopora damicornis*	17-Jan-14	22.42	0.04
Rottnest Island - *Pocillopora damicornis*	17-Jan-14	23.69	0.03
Rottnest Island - *Pocillopora damicornis*	17-Jan-14	22.52	0.05
Rottnest Island - *Pocillopora damicornis*	27-Feb-14	23.09	0.04
Rottnest Island - *Pocillopora damicornis*	27-Feb-14	23.51	0.04

Rottnest Island - *Pocillopora damicornis*	27-Feb-14	22.53	0.06
Rottnest Island - *Pocillopora damicornis*	27-Feb-14	22.79	0.05
Rottnest Island - *Pocillopora damicornis*	14-May-14	22.12	0.05
Rottnest Island - *Pocillopora damicornis*	14-May-14	22.39	0.05
Rottnest Island - *Pocillopora damicornis*	14-May-14	21.82	0.06
Rottnest Island - *Pocillopora damicornis*	14-May-14	21.88	0.04
Rottnest Island - *Pocillopora damicornis*	18-Jul-14	24.01	0.05
Rottnest Island - *Pocillopora damicornis*	18-Jul-14	23.83	0.04
Rottnest Island - *Pocillopora damicornis*	18-Jul-14	24.06	0.05
Rottnest Island - *Pocillopora damicornis*	18-Jul-14	23.22	0.05
Rottnest Island - *Pocillopora damicornis*	02-Sep-14	23.82	0.06
Rottnest Island - *Pocillopora damicornis*	02-Sep-14	23.83	0.07
Rottnest Island - *Pocillopora damicornis*	02-Sep-14	24.34	0.05
Rottnest Island - *Pocillopora damicornis*	02-Sep-14	24.04	0.06
Rottnest Island - *Pocillopora damicornis*	24-Oct-14	24.20	0.06
Rottnest Island - *Pocillopora damicornis*	24-Oct-14	23.02	0.05
Rottnest Island - *Pocillopora damicornis*	24-Oct-14	23.03	0.06
Rottnest Island - *Pocillopora damicornis*	24-Oct-14	23.00	0.06
Rottnest Island - *Pocillopora damicornis*	19-Dec-14	22.63	0.10
Rottnest Island - *Pocillopora damicornis*	19-Dec-14	23.44	0.14
Rottnest Island - *Pocillopora damicornis*	19-Dec-14	23.36	0.18
Rottnest Island - *Pocillopora damicornis*	19-Dec-14	23.53	0.15
Rottnest Island - *Pocillopora damicornis*	31-Mar-15	22.91	0.15
Rottnest Island - *Pocillopora damicornis*	31-Mar-15	23.02	0.08
Rottnest Island - *Pocillopora damicornis*	31-Mar-15	22.40	0.11
Rottnest Island - *Pocillopora damicornis*	31-Mar-15	23.11	0.11
